# Translational Activation of ATF4 through Mitochondrial Anaplerotic Metabolic Pathways Is Required for DLBCL Growth and Survival

**DOI:** 10.1158/2643-3230.BCD-20-0183

**Published:** 2021-11-09

**Authors:** Meng Li, Matthew R. Teater, Jun Young Hong, Noel R. Park, Cihangir Duy, Hao Shen, Ling Wang, Zhengming Chen, Leandro Cerchietti, Shawn M. Davidson, Hening Lin, Ari M. Melnick

**Affiliations:** 1 Department of Medicine, Division of Hematology & Medical Oncology, Weill Cornell Medicine, New York, New York.; 2 Department of Chemistry and Chemical Biology, Howard Hughes Medical Institute, Cornell University, Ithaca, New York.; 3 Lewis-Sigler Institute for Integrative Genomics, Princeton University, Princeton, New Jersey.; 4 Division of Biostatistics and Epidemiology, Weill Cornell Medicine, New York, New York.

## Abstract

**Abstract:**

Diffuse large B-cell lymphomas (DLBCL) are broadly dependent on anaplerotic metabolism regulated by mitochondrial SIRT3. Herein we find that translational upregulation of ATF4 is coupled with anaplerotic metabolism in DLBCLs due to nutrient deprivation caused by SIRT3 driving rapid flux of glutamine into the tricarboxylic acid (TCA) cycle. SIRT3 depletion led to ATF4 downregulation and cell death, which was rescued by ectopic ATF4 expression. Mechanistically, ATF4 translation is inhibited in SIRT3-deficient cells due to the increased pools of amino acids derived from compensatory autophagy and decreased glutamine consumption by the TCA cycle. Absence of ATF4 further aggravates this state through downregulation of its target genes, including genes for amino acid biosynthesis and import. Collectively, we identify a SIRT3–ATF4 axis required to maintain survival of DLBCL cells by enabling them to optimize amino acid uptake and utilization. Targeting ATF4 translation can potentiate the cytotoxic effect of SIRT3 inhibitor to DLBCL cells.

**Significance:**

We discovered the link between SIRT3 and ATF4 in DLBCL cells, which connected lymphoma amino acid metabolism with ATF4 translation via metabolic stress signals. SIRT3–ATF4 axis is required in DLBCL cells regardless of subtype, which indicates a common metabolic vulnerability in DLBCLs and can serve as a therapeutic target.

*This article is highlighted in the In This Issue feature, p. 1*

## Teaser

ATF4 expression is required for DLBCL proliferation and is induced by rapid consumption of amino acids. Depletion of SIRT3 suppresses the TCA cycle and impairs ATF4 translation by increasing amino acid pools generated through autophagy, leading to cell death.

## INTRODUCTION

Diffuse large B-cell lymphomas (DLBCL) are highly proliferative and aggressive tumors that mostly arise from germinal center (GC) B cells or post–GC B cells. Critical to their survival, DLBCLs must tolerate various forms of cellular stress associated with their rapid proliferation and depletion of nutrients in their immediate microenvironment (1). To overcome these challenges, DLBCLs undergo metabolic reprogramming to support efficient energy and metabolic precursor production, which can be accomplished in part through induction and constitutive activation of adaptive stress responses/signals (1, 2). Stress survival pathways constitute an important source of non-oncogene addiction to specific proteins. Understanding mechanisms through which such non-oncogene dependencies contribute to lymphomagenesis can point to tumor vulnerabilities amenable to therapeutic intervention. Moreover, targeting such stress mechanisms can be broadly relevant to DLBCLs regardless of their genetic heterogeneity and spectrum of somatic mutations (1). This is important because approximately 40% of patients with DLBCL are not cured with current therapies (3).

Mitochondria are critically important to tumor-associated metabolic reprogramming processes, working as metabolic hubs to support cell growth and proliferation, and as sensors of intracellular stresses that could threaten survival (4, 5). SIRT3, a member of the Sirtuin family of proteins, resides within mitochondria and regulates mitochondrial metabolic functions through its lysine deacetylase activity (6–8). SIRT3 was recently shown to function as a critical source of non-oncogene addiction in DLBCL, due primarily to its role in driving efficient glutamine entry into the tricarboxylic acid (TCA) cycle (9). TCA cycle–mediated generation of metabolic biosynthetic precursors was profoundly impaired in SIRT3-deficient DLBCL cells and led to activation of autophagy, which in turn induced proliferation arrest and cell death (9, 10). SIRT3 deficiency impaired lymphomagenesis *in vivo*, and its expression was shown to be aberrantly induced in primary human DLBCLs and linked to inferior clinical outcomes (9).

The critical role of SIRT3 in supporting the massive metabolic needs of DLBCL cells points to the question of how SIRT3 is positioned among cellular pathways involved in nutrient and proliferative stress responses. SIRT3 functions are often cell-context dependent (11). For example, SIRT3 was implicated as a tumor suppressor in solid tumor cells such as breast and ovarian cancer cell lines (12, 13). In those cells, loss of SIRT3 impaired mitochondrial metabolism, including the TCA cycle, and led to accumulation of reactive oxygen species. In turn, the elevated oxidative stress led to hypoxia-induced factor 1α (HIF1α) stabilization, which can transduce hypoxia signals to the cell nucleus, with corresponding changes in transcriptional programming (13). Thus, shifts in metabolic pathways can induce transcriptional changes to support adaptation of cells to micronutrient availability and meet their growth requirements.

In contrast, SIRT3 promotes tumorigenesis in DLBCL, where it is required for glutamine-fueled anaplerosis, and where its loss of function leads to destructive autophagy (9). Most critically, it is not known why SIRT3-deficient DLBCL cells are so vulnerable to such metabolic changes and autophagy, which points to potentially novel and critical nutrient regulatory circuits occurring in this disease. Here, we set out to identify downstream signals induced by SIRT3 deficiency in DLBCL cells to gain insight into how SIRT3 could interface with nutrient flux stress response pathways to support proliferation of DLBCL cells and promote tumorigenesis in this particular tumor context.

## RESULTS

### ATF4 but Not HIF1a Target Genes Are Downregulated after *SIRT3* Knockdown in DLBCL Cells

**Figure 1.**
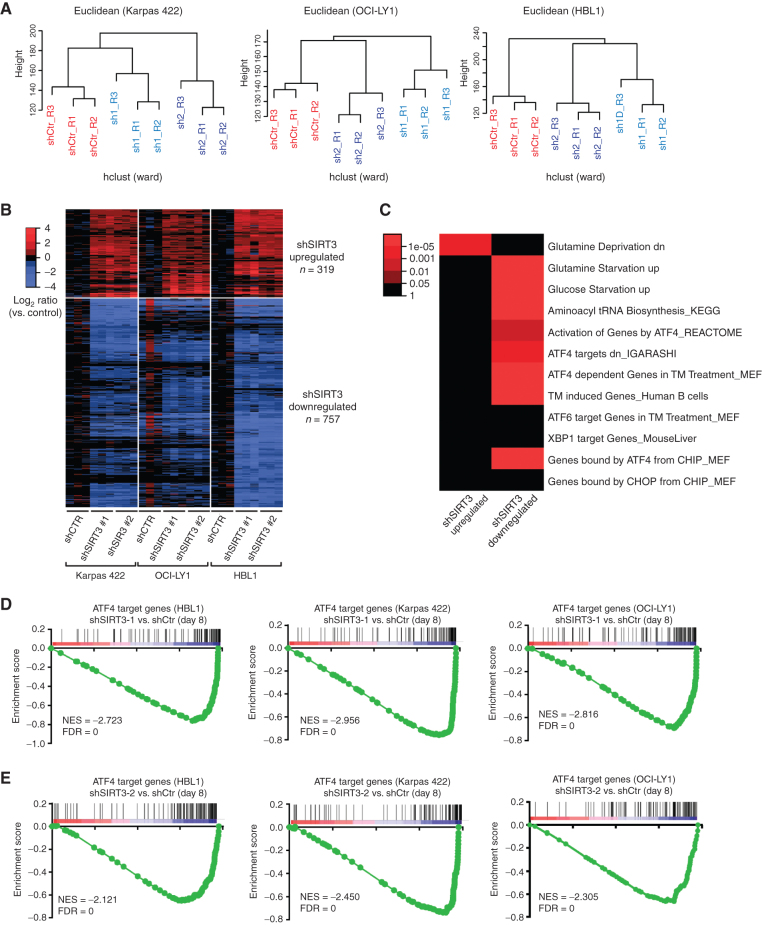
Knocking down SIRT3 caused ATF4 signaling inhibition but not HIF1a. **A,** Dendrograms from hierarchical clustering of RNA-seq data from three DLBCL cells lines transduced with lentiviruses containing control (scramble) or two SIRT3 shRNAs. **B,** Heatmap showing differential expression in SIRT3 knockdown cells versus control (FC > 1.5, *q* < 0.05). **C,** Heatmap showing enrichment of SIRT3 knockdown signatures within key pathways. CHIP, chromatin immunoprecipitation; CHOP, C/EBP homologous protein; dn, down; KEGG, Kyoto Encyclopedia of Genes and Genomes; MEF, mouse embryonic fibroblast; TM, tunicamycin. **D,** GSEA (51, 52) showing the enrichment of ATF4 target genes in SIRT3-downregulated genes in Karpas 422, OCI-LY1, and HBL1 cells with SIRT3 sh1 versus control scramble shRNAs. The rank lists were from RNA-seq analysis from **B**. ATF4 target genes were summarized from previous publications (17, 18). **E,** GSEA (51, 52) showing the enrichment of ATF4 target genes in SIRT3-downregulated genes in Karpas 422, OCI-LY1, and HBL1 cells with SIRT3 sh2 versus control scramble shRNAs. The rank lists were from RNA-seq analysis from **B**. The same ATF4 target gene list was used here as in **D**.

Mitochondrial signals drive transcriptional programming of cells through various mechanisms including cross-talk with transcription factors, chromatin-modifying complexes, or modulating the abundance of precursor molecules utilized for posttranslational modifications of histones (4, 5, 14). To explore pathways downstream of SIRT3 in an unbiased fashion, we performed RNA sequencing (RNA-seq) in three cell lines corresponding to OXPHOS (oxidative phosphorylation gene expression high: Karpas 422), GCB (GC B-cell like: OCI-LY1), and ABC (activated B-cell like: HBL1) DLBCL subtypes (15). SIRT3 was previously shown to be required for proliferation and survival of these cells (9). SIRT3 knockdown was performed in replicates in each cell line, using two different SIRT3 short hairpin RNAs (shRNA) and control shRNA. Unsupervised analysis revealed that SIRT3 depletion induces distinct transcriptional profiles in these cells (Fig. 1A). We found that DLBCL cells clustered together according to shRNA hairpins, indicating consistent transcriptional effects following SIRT3 knockdown. Using a supervised analysis, we identified 1,075 differentially regulated genes [fold change (FC) > 1.5, *q* < 0.05] in common across all three cell lines (Fig. 1B; Supplementary Table S1). Of these, 756 were downregulated and 319 upregulated upon SIRT3 depletion. Performing pathway enrichment analysis, we observed significant upregulation of genes that are normally downregulated by glutamine deprivation (Fig. 1C). Reciprocally, there was downregulation of genes that are induced by glutamine and glucose starvation as well as genes involved in aminoacyl-tRNA biogenesis. There was also significant downregulation of endoplasmic reticulum (ER) stress genes induced by tunicamycin, including a subset of tunicamycin-induced genes that are dependent on ATF4. Many of these downregulated pathways are related to the transcription factor activated transcriptional factor 4 (ATF4), which is known to be induced by integrated stress response, such as ER stress or amino acid deprivation (16). Several additional ATF4 target gene sets defined by chromatin immunoprecipitation (ChIP) sequencing or functional assays (17, 18) were also specifically downregulated in SIRT3-deficient DLBCL cells (Fig. 1C). However, transcriptional programs linked to other ER stress transcription factors such as ATF6 (19), XBP1 (20), and CHOP (17) were not perturbed by SIRT3 knockdown (Supplementary Fig. S1A). To further confirm a robust connection between SIRT3 and ATF4, we performed gene set enrichment analysis (GSEA) using the ranked gene expression profiles of each individual cell line and again observed highly significant depletion of ATF4 target genes in all three DLBCL types [normalized enrichment score (NES) < −2.7 and FDR = 0; Fig. 1D and E].

Previous reports indicated that *Sirt3* can attenuate the function of HIF1α and its downstream transcriptional effects in epithelial tumor cells (12, 13). Given that HIF1α has oncogenic roles in multiple cancer types, we wondered whether HIF1α might be modulated by SIRT3 in DLBCLs as well. In contrast to ATF4, there was no enrichment and instead mostly a trend toward depletion of HIF1α target genes in SIRT3 knockdown DLBCL cells (Supplementary Fig. S1B). HIF1α stability was enhanced by loss of SIRT3 under hypoxic conditions in two transformed adherent epithelial cell lines (HEK-293T and HCT116), consistent with previous reports in solid tumor cells (refs. 12, 13; Supplementary Fig. S1C). In contrast, we did not observe accumulations of HIF1α protein after SIRT3 knockdown in either normoxic (21%) or hypoxic (1%) conditions in DLBCL cell lines (Supplementary Fig. S1D). Therefore, SIRT3 regulation of HIF1 appears to be context dependent, while SIRT3 signals through different mechanisms in DLBCL cells.

### SIRT3 Deficiency Suppresses ATF4 Translation but Not Transcription

**Figure 2.**
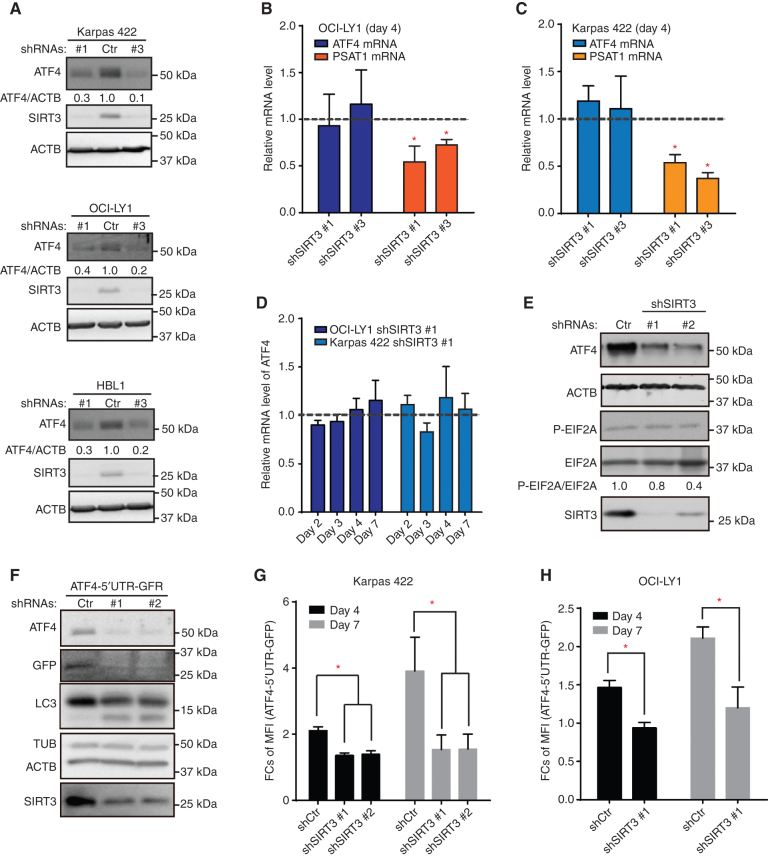
Knockdown SIRT3 caused ATF4 protein decrease via translation regulation. **A,** Western blots show ATF4 protein levels in different DLBCL cells with control or SIRT3 shRNAs. SIRT3 was blotted showing knockdown efficiency, and ACTB was used as reference protein control. **B,** qPCR results reflect the mRNA levels of ATF4 and PSAT1 in OCI-LY1 cells. Samples were harvested at day 4 after viral transduction. Results were normalized to the mRNA levels in control shRNA–transduced cells. **C,** qPCR results reflect the mRNA levels of ATF4 and PSAT1 in Karpas 422 cells. Samples were harvested at day 4 after viral transduction. Actin mRNA was used as reference, and results were normalized to the mRNA levels in control shRNA–transduced cells. **D,** qPCR results show the relative levels of ATF4 mRNAs in different cell lines at different time points after shRNA transduction. Samples were harvested at days 2, 3, 4, and 7 after viral transduction. Actin mRNA was used as reference, and results were normalized to the mRNA levels in control shRNA–transduced cells. **E,** Western blots show changes of phosphorylation of EIF2A and ATF4 protein levels in Karpas 422 cells with control or SIRT3 shRNAs. Total EIF2A and ACTB were blotted as loading controls. **F,** Western blots show GFP expression from the ATF4-5′UTR-GFP reporter and endogenous ATF4 protein levels in Karpas 422 cells with control or SIRT3 shRNAs. Tubulin and ACTB were blotted as loading controls. **G,** FCs of mean fluorescence intensity (MFI) of GFP expressed from the ATF4-5′UTR-GFP translation reporter in Karpas 422 cells with control or SIRT3 shRNAs. The data were collected from days 4 and 7 after viral transduction. MFI of normal yellow fluorescent protein (YFP) expression in control or SIRT3 knockdown cells were used for normalization to avoid background translation variations. **H,** FCs of MFI of GFP expressed from the ATF4-5′UTR-GFP translation reporter in OCI-LY1 cells with control or SIRT3 shRNAs. The data were collected from days 4 and 7 after viral transduction. MFI of normal YFP expression in control or SIRT3 knockdown cells were used for normalization to avoid background translation variations. Error bars represent the mean ± SD of three or more replicates.

Our transcriptional profiling studies pointed to ATF4 being regulated by SIRT3 in DLBCL cells. Therefore, we next examined ATF4 expression after SIRT3 depletion in DLBCL cells. SIRT3 knockdown with two independent shRNAs resulted in reduction of ATF4 protein abundance in five independent DLBCL cell lines regardless of subtype (Fig. 2A; Supplementary Fig. S2A). However, ATF4 mRNA abundance did not change in qPCR assays of DLBCL cells with SIRT3 shRNAs (Fig. 2B and C). In contrast, there was significant transcriptional downregulation of the ATF4 target gene *PSAT1* in the same experiments, consistent with the reduction in ATF4 protein and downregulation of the ATF4 transcriptional program. After SIRT3 knockdown, ATF4 mRNA abundance remained stable even when measured at different time points (Fig. 2D).

ATF4 protein levels are reported to be regulated through translational mechanisms under stress conditions (16). The ATF4 mRNA contains a 5′-UTR (untranslated region) region with various regulatory elements responsive to translational control (21) by eukaryotic translation initiation factor 2A (EIF2A). Without stress, EIF2A is not phosphorylated and ATF4 translation is low; under stress, EIF2A is phosphorylated on Ser51 and ATF4 translation is activated. Despite this, there was no obvious difference in EIF2A phosphorylation after SIRT3 knockdown (Fig. 2E). To further explore its translational control, we employed a fluorescent reporter for ATF4 translation, generated by fusing the ATF4 5′-UTR with a GFP open reading frame (ATF4-5′UTR-GFP; ref. 22). To validate the reporter in our lymphoma system, we performed Western blots in DLBCL cells expressing the ATF4-5′UTR-GFP after induction of SIRT3 or control shRNA. Immunoblotting for either endogenous ATF4 or for GFP expressed from the ATF4-5′UTR-GFP reporter showed that both proteins were strongly downregulated by SIRT3 depletion (Fig. 2F; Supplementary Fig. S2B), confirming the suitability of this construct to reflect ATF4 protein regulation. Using this reporter system, we confirmed that ATF4 translation was inhibited in both Karpas 422 and OCI-LY1 cells (Supplementary Fig. S2C) at day 4 and day 7 after SIRT3 knockdown using flow cytometry (Fig. 2G and H). Thus, SIRT3 drives ATF4 protein expression through translational regulation, and loss of SIRT3 impairs ATF4 protein translation with consequent downregulation of ATF4 target genes.

### ATF4 Is Required for DLBCL Proliferation and Contributes to the Oncogenic Effects of SIRT3 in DLBCL Cells

**Figure 3.**
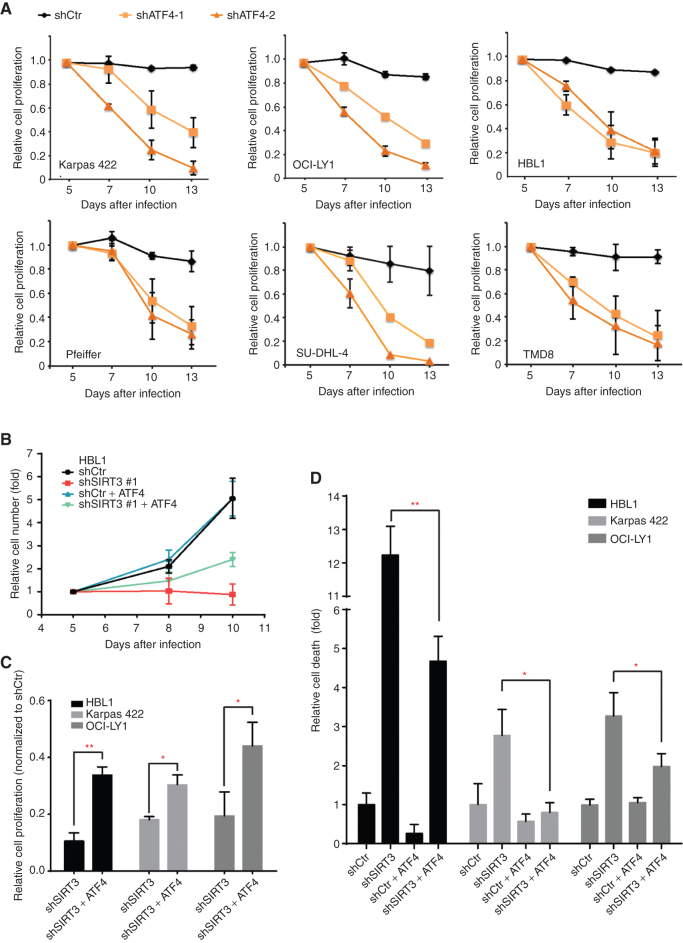
ATF4 is required in DLBCL cells and is partially responsible for SIRT3′s functions to promote DLBCL cell proliferation and survival. **A,** Effect of ATF4 knockdown on the proliferation of DLBCL cell lines. Each cell line was infected with lentivirus expressing control or ATF4 shRNAs and yellow fluorescent protein (YFP), and YFP^+^ viable (DAPI^−^) cells were monitored by flow cytometry for 8 days. **B,** FCs of cell numbers of HBL1 cells expressing control or SIRT3 shRNA with or without exogeneous ATF4. HBL1 cells were transduced with viral vectors containing shRNAs or genes as presented. Cell number changes were normalized to data of initial time point (day 3 after infection). **C,** Summarized results show the rescue effects of exogenous ATF4 to SIRT3 shRNA-induced cell proliferation inhibition in different DLBCL cells. The data were summarized from day 10 after infections and normalized to the cell numbers of their respective control shRNA–expressing cell. **D,** Effects of exogenous ATF4 on different DLBCL cells expressing control or SIRT3 shRNAs. Dead cells were stained with DAPI and quantified through flow cytometry. The relative cell death was calculated by normalizing the percentage of dead cells in control shRNA–expressing cells in respective cell lines. *, *P* < 0.05; **, *P* < 0.01. Error bars represent the mean ± SD of three or more replicates.

Because SIRT3 knockdown leads to proliferation arrest and apoptosis of DLBCL cells, we wondered whether this could be attributed to the reduction in ATF4 levels. To determine whether this might be the case, we first performed knockdown studies using two independent ATF4 shRNAs in a panel of DLBCL cell lines (Supplementary Fig. S3A). Notably, ATF4 depletion markedly impaired proliferation across DLBCL cell lines regardless of disease subtype (Fig. 3A), indicating their dependency on sustained ATF4 expression. To explore to what extent reduction of ATF4 contributes to the antilymphoma effect of SIRT3 depletion, we performed rescue experiments with exogeneous ATF4 in SIRT3-deficient DLBCL cells. We first compared inhibition of cell proliferation by SIRT3 shRNA with or without ATF4 expression. Consistent with prior reports (9), SIRT3 shRNA completely blocked cell proliferation after 5 days of culture, whereas ectopic expression of ATF4 alone had no effect (Fig. 3B). However, ectopic expression of ATF4 partially rescued the proliferation arrest induced by SIRT3 depletion (Fig. 3B; Supplementary Fig. S3B). This effect was validated in two additional DLBCL cell lines (Karpas 422 and OCI-LY1; Fig. 3C; Supplementary Fig. S3B). SIRT3 knockdown was also shown to induce cell death in DLBCL cells (9). This effect was also partially rescued by ectopic ATF4 expression in DLBCL cells after SIRT3 knockdown (Fig. 3D; Supplementary Fig. S3C). Exogenous ATF4 expression was higher than endogenous protein and was not affected by SIRT3 shRNAs (Supplementary Fig. S3D) because the cDNA of exogenous ATF4 does not contain its regulatory 5′UTR region. Collectively, these data point to a critical role of ATF4 in maintaining proliferation and survival of DLBCL cells downstream of SIRT3.

### ATF4 Expression Is Associated with SIRT3 Levels in Primary Lymphoma Cells

**Figure 4.**
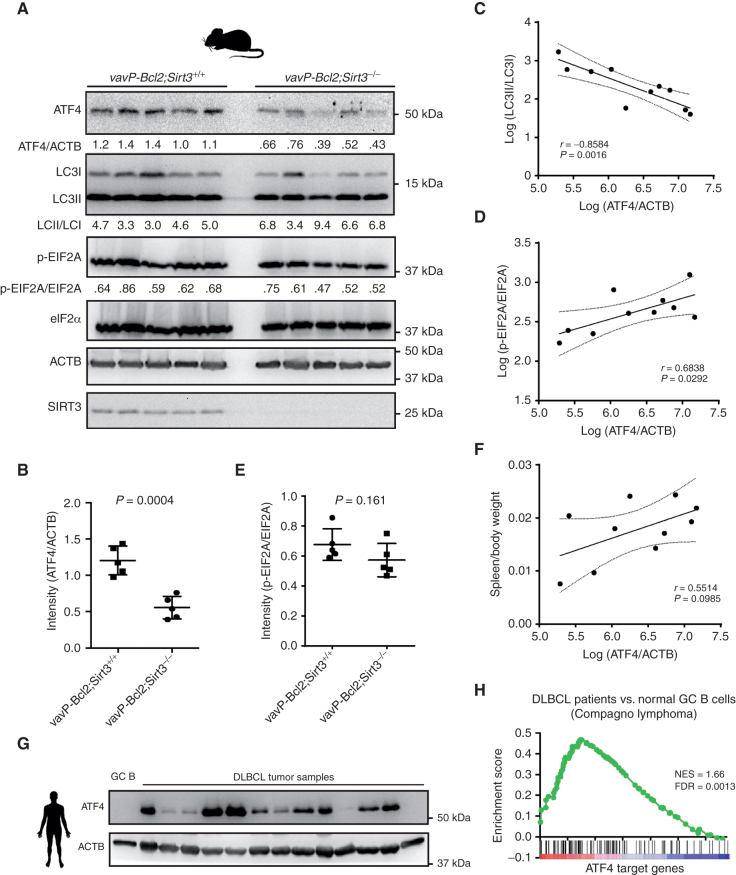
ATF4 protein level is decreased in *vavP-Bcl2;Sirt3*^−/−^ mice and associated with lymphoma progression. **A,** Western blot results show the protein levels of ATF4, LC3, EIF2A, ACTB, and SIRT3 in splenocytes from *vavP-Bcl2;Sirt3*^+/+^ and *vavP-Bcl2;Sirt3*^−/−^ mice. The protein amounts were quantified with densitometry results. **B,** Summarized results of ATF4 protein level normalized to ACTB in splenocytes from *vavP-Bcl2;Sirt3*^+/+^ and *vavP-Bcl2;Sirt3*^−/−^ mice. The protein amounts were quantified with densitometry results from Western blots. **C,** Correlation between levels of autophagy (LC3II/LC3I) and ATF4 (ATF4/ACTB) in splenocytes from *vavP-Bcl2;Sirt3*^+/+^ and *vavP-Bcl2;Sirt3*^−/−^ mice. The data for correlation study were obtained with densitometry results from Western blots. **D,** Correlation between levels of phospho-EIF2A (p-EIF2A/EIF2A) and ATF4 (ATF4/ACTB) in splenocytes from *vavP-Bcl2;Sirt3*^+/+^ and *vavP-Bcl2;Sirt3*^−/−^ mice. The data for correlation study were obtained with densitometry results from Western blots. **E,** Summarized results of phospho-EIF2A level normalized to total EIF2A in splenocytes from *vavP-Bcl2;Sirt3*^+/+^ and *vavP-Bcl2;Sirt3*^−/−^ mice. The protein amounts were quantified with densitometry results from Western blots. **F,** Correlation between splenomegaly phenotype (spleen/body weight) and levels of ATF4 (ATF4/ACTB) in splenocytes from *vavP-Bcl2;Sirt3*^+/+^ and *vavP-Bcl2;Sirt3*^−/−^ mice. ATF4 levels were quantified with densitometry results from Western blots. **G,** Western blot results show ATF4 levels from human DLBCL tumor samples or normal GC B cells from human tonsil. ACTB levels were used as loading control. **H,** GSEA shows the enrichment of ATF4 target genes in DLBCL tumors versus normal GC B cells. Gene expression data were from published microarray data (53). Error bars represent the mean ± SD of three or more replicates.

Sirt3 knockout was reported to impair lymphomagenesis in *VavP-Bcl2* mice, which was associated with a reduction in TCA cycle intermediates as well as induction of autophagy (9). Given our data showing that SIRT3 is required to maintain ATF4 expression in DLBCL cell lines, we next examined whether this was also the case in primary lymphomas developing *in vivo*. Using Western blots, we showed that indeed ATF4 protein levels were significantly decreased in *VavP-Bcl2;Sirt3*^−/−^ versus *VavPBcl2;Sirt3*^WT^ primary lymphomas (Fig. 4A and B). We also examined the degree of autophagy in these tumors by determining their LC3II and LC3I ratios by Western blot analysis, which confirmed higher levels of autophagy in *Sirt3*^−/−^ lymphomas (Supplementary Fig. S4A). Further analysis revealed a strong and significant negative correlation between autophagy (LC3II/LC3I ratios) and ATF4 (ATF4/ACTB) level (Fig. 4C), suggesting that autophagy may be linked to suppression of ATF4. Finally, although ATF4 protein showed positive correlation with the relative abundance of phospho-EIF2A (p-EIF2A/EIF2A; Fig. 4D), the phospho-EIF2A levels only showed the trend of decrease in *Sirt3*^−/−^ versus *Sirt3*^WT^ lymphomas cells but was not statistically significant (Fig. 4E). Splenomegaly is an indicator of tumor burden, and we observed generally positive correlation between ATF4 protein abundance and degree of splenomegaly (Fig. 4F). DLBCLs arise from GC B cells, which are highly self-limited in their replicative capacity (23). Western blots performed in primary human DLBCLs and GC B cells revealed generally high levels of ATF4 in the lymphomas versus their normal B-cell counterparts (Fig. 4G). Accordingly, ATF4 target genes were also significantly enriched in human DLBCL tumors compared with normal GC B cells (Fig. 4H), as well as when comparing DLBCL cell lines with GC B cells (Supplementary Fig. S4B). Hence, ATF4 is aberrantly expressed in primary murine and human lymphomas, where it appears to be linked to SIRT3 expression and inversely correlated with autophagy.

### ATF4 Protein Levels Are Controlled through a SIRT3–GDH–TCA–Autophagy–ATF4 Cascade

**Figure 5.**
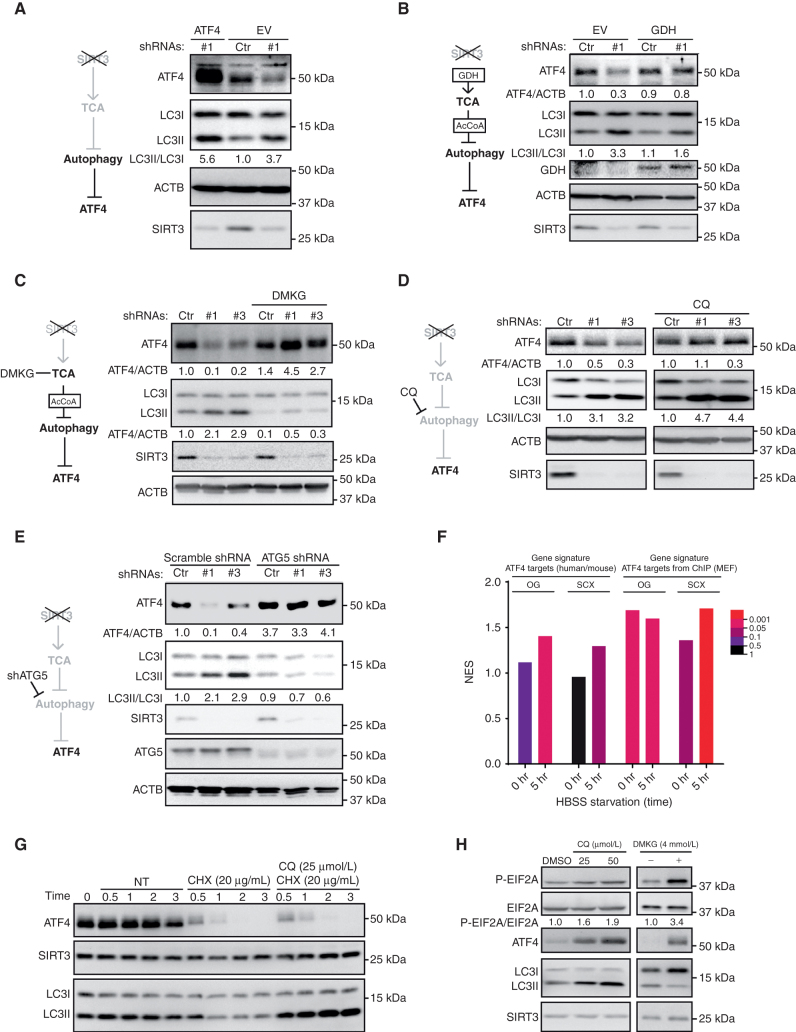
ATF4 protein level is regulated downstream of the SIRT3–GDH–TCA cycle–autophagy cascade. **A,** Western blots show the effects of ATF4 expression to autophagy activation induced by SIRT3 shRNA in Karpas 422 cells. Protein level changes were quantified with densitometry results. Hypothetical cascade model is presented to the left. EV, empty vector. **B,** Western blots show the ATF4 protein level being rescued by exogenous GDH in SIRT3 knockdown Karpas 422 cells. Protein level changes were quantified with densitometry results. Hypothetical cascade model is presented to the left. **C,** Western blots show the ATF4 protein level being rescued by DMKG in SIRT3 knockdown Karpas 422 cells. Protein level changes were quantified with densitometry results. Hypothetical cascade model is presented to the left. **D,** Western blots show the ATF4 protein level being rescued by CQ (50 μmol/L) in SIRT3 knockdown Karpas 422 cells. CQ treatment was done in 16 to 18 hours, followed by immunoblot with the indicated antibodies. Densitometry values are shown for ATF4/ACTB and LC3II/LC3I ratios. Hypothetical cascade model is presented to the left. **E,** Western blots show the changes of ATF4 protein level control or ATG5 knockdown Karpas 422 cells with control or SIRT3 shRNAs. Protein level changes were quantified with densitometry results. Hypothetical cascade model is presented to the left. **F,** Summarized bar plot shows the NES (*y*-axis) and FDRs (bar colors) of results from GSEA using published proteomic data (24). NES show the enrichment of ATF4 target genes (human and mouse) or ATF4 target genes from ChIP (mouse embryonic fibroblasts, MEF) in Atg5 knockout MEF cells in normal or starvation (5 hours) treatment condition. The experiments were done with stable isotope labeling by amino acids in cell culture (SILAC) coupled with off-gel fractionations (OG) and strong cation exchange chromatography (SCX) methods. HBSS, Hank's Balanced Salt Solution. **G,** Western blots show ATF4 protein levels under CHX and CQ treatment in Karpas 422 cells. Protein samples were collected at the indicated time points after treatment to monitor the kinetics of ATF4 degradation. SIRT3 and LC3 were blotted as controls. NT, not treated. **H,** EIF2A phosphorylation from Karpas 422 cells exposed to two different doses of CQ or DMSO, or to DMKG treatment. Proteins were blotted with the indicated antibodies, and the densitometry showed relative abundance of phospho-EIF2A over total EIF2A.

SIRT3 mediates proliferation and survival in DLBCL by driving glutamine into the TCA cycle through enhanced GDH activity, which in turn leads to increased production of metabolic precursors such as acetyl-CoA (AcCoA) and prevents destructive autophagy—thus delineating a crucial SIRT3–GDH–TCA cycle–autophagy pathway (9). Following this pathway in DLBCL cells by Western blot analysis after SIRT3 knockdown confirmed that reduction of ATF4 was accompanied by induction of autophagy as shown by LC3II/LC3I ratios as well as reduction of histone acetylation, which reflects impaired production of AcCoA from the TCA cycle (ref. 14; Supplementary Fig. S5A). To determine the relative position of ATF4 in this critical SIRT3-regulated pathway, we next tested whether exogeneous ATF4 could rescue the induction of autophagy induced by SIRT3 knockdown. Remarkably, even though exogenous ATF4 was expressed robustly, there was no rescue of autophagy activation induced by loss of SIRT3 (Fig. 5A). These data place suppression of ATF4 downstream of autophagy activation in SIRT3-deficient DLBCL cells.

To further support this notion, we attempted to prevent ATF4 downregulation through rescue of the SIRT3 pathway at various, more upstream stages. GDH is the key direct deacetylation substrate of SIRT3 in DLBCL cells, and ectopic expression of this enzyme can rescue autophagy and proliferation arrest due to SIRT3 depletion (9). Here we show that ectopic expression of GDH also rescues ATF4 protein expression in DLBCL cells and prevents strong induction of autophagy (Fig. 5B). Downstream of GDH, the effects of SIRT3 depletion in DLBCL cells can also be rescued by administration of exogenous sources of α-KG (such as DMKG, dimethyl-α-Ketoglutarate) that bypass GDH to directly enter the TCA cycle (9). Notably we found that DMKG prevented autophagy activation, generation of LC3II, and ATF4 downregulation in DLBCL cells transduced with SIRT3 shRNA (Fig. 5C), which suggest that the metabolic signals from the TCA cycle may help sustain ATF4 protein level.

Autophagy occurs downstream of TCA cycle impairment after SIRT3 knockdown and is not rescued by ATF4, while DMKG blocks autophagy and rescues ATF4 protein reduction in SIRT3-depleted cells. Therefore, it is likely that ATF4 was regulated downstream of autophagy induced by SIRT3 depletion. To test this, we used two autophagy inhibitors, chloroquine (CQ) and bafilomycin A1 (BafA1), to inhibit the late stage of autophagy, lead to accumulation of LC3II, and rescue the reduction of ATF4 in SIRT3-depleted cells (Fig. 5D; Supplementary Fig. S5B). Furthermore, we suppressed autophagy by knocking down the critical autophagy gene, for example, *ATG5*, a critical autophagy gene for the autophagosome formation. DLBCL cells with ATG5 shRNAs manifested impaired autophagy and resistance to SIRT3 knockdown (9). Here we observed that ATG5-depleted DLBCL cells also showed some resistance to the inhibition of ATF4 by SIRT3 knockdown (Fig. 5E). As expected, ATG5 depletion also impaired autophagy (LC3II/LC3I ratios) induced by SIRT3 shRNA. The relation between autophagy and ATF4 may not be limited to DLBCL cells, because our reanalysis of proteomic profiles generated in ATG5 knockout mouse embryonic fibroblasts (MEF) by Robin and colleagues (24) generally showed significant upregulation of proteins encoded by ATF4 target genes (Fig. 5F; Supplementary Fig. S5C–S5F). This effect was further enhanced in starvation condition, consistent with the known responsiveness of ATF4 to nutrient signaling (Fig. 5F).

Given these results, we next considered how autophagy might regulate ATF4 expression. Many proteins (e.g., p62) are degraded through autophagy-mediated lysosomal degradation (25). To explore whether autophagy induces ATF4 protein degradation, we evaluated its half-life in cycloheximide (CHX)-treated cells and observed that the protein is extremely rapidly degraded in DLBCL cells, essentially disappearing within 2 hours after CHX blockade. However, ATF4 stability was not enhanced (Fig. 5G; Supplementary Fig. S5G) when we used CQ to block autophagy-related lysosomal protein degradation (26). In contrast, the proteasome inhibitor MG132 did maintain ATF4 protein stability in CHX-treated cells (Supplementary Fig. S5H). Though autophagy does not control ATF4 protein level directly, we observed that both DMKG and CQ can block autophagy at early and late stages, respectively, and induced the phosphorylation of EIF2A at serine 51 (Fig. 5H). This finding shows that autophagy negatively regulates ATF4 translation, which is consistent with the effect of SIRT3 depletion in DLBCL cells.

Collectively these data indicate that ATF4 downregulation is dependent on induction of autophagy and functions downstream of the SIRT3–GDH–TCA–autophagy cascade, where its translational regulatory state represents a critical vulnerability to DLBCL cells.

### ATF4 Accumulation Is a Response to Amino Acid Flux Regulated by SIRT3 and Autophagy

**Figure 6.**
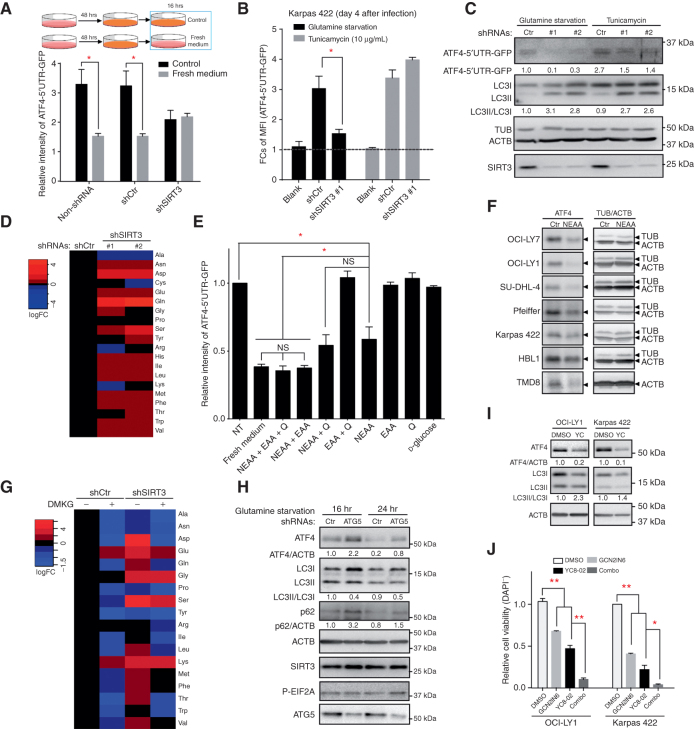
ATF4 translation and protein level respond to nutrient level and are regulated by autophagy. **A,** FCs of mean fluorescence intensity (MFI) of the GFP reporter expressed from the ATF4-5′UTR-GFP translation reporter in Karpas 422 cells containing control or SIRT3 shRNAs under different culture conditions. Top, experiments were done as in the schema. Briefly, cells were cultured with fresh medium for 48 hours and then replenished with same volume of fresh medium or maintained without replenishment (as a control) for another 16 hours. The *y*-axis denotes GFP signal intensity relative to control cells without expression of reporter, determined with MFI of GFP signals from flow cytometer. **B,** FCs of MFI of the GFP reporter expressed from the ATF4-5′UTR-GFP translation reporter in Karpas 422 cells with control or SIRT3 shRNAs under control or glutamine starvation or tunicamycin (10 μg/mL) treatment. Both starvation and tunicamycin treatment were maintained for 15 hours at day 4 after viral transduction. **C,** Western blot results show the GFP expression from the ATF4-5′UTR-GFP translation reporter in Karpas 422 cells from **B**. **D,** The heatmap shows that relative abundances of amino acids were detected by metabolic profiling from Karpas 422 cells transduced with SIRT3 or control shRNAs. The metabolite levels were mean value from five to six replicate samples obtained on day 6 after infection. **E,** Relative activities of the ATF4-5′UTR-GFP reporter in Karpas 422 were cultured for 48 hours, replenished with fresh media or with the indicated nutrients, and then assessed for ATF4 translation reporter activity 16 hours later. The *y*-axis denotes GFP signal intensity relative to control cells without expression of reporter, determined with MFI of GFP signals from flow cytometer. NT, not treated; Q, glutamine. **F,** Western blots show ATF4 protein levels from the indicated cell lines cultured as in **A** and **E**, and then replenished with NEAA or not replenished, after which immunoblots were performed for ATF4, with tubulin and actin as loading controls, in control or NEAA-added conditions. **G,** The heatmap shows the amino acid abundance measured with LC/MS from Karpas 422 cells transduced with SIRT3 or control shRNA and cultured with or without DMKG supplementation. The values show the average logFC from three replicates in amino acid abundance as compared with DSMO-treated control shRNA–transduced cells. **H,** Western blot results show ATF4 level changes in control or ATG5 knockdown Karpas 422 cells under glutamine starvation condition. Samples were collected at different time points, and protein level changes were quantified with densitometry results. **I,** Western blots show the ATF4 and autophagy changes in Karpas 422 cells treated with DMSO or YC8-02 (YC; 3 μmol/L) for 40 hours. Cell lysates were subjected to Western blot using the indicated antibodies. Protein levels were quantified with densitometry results. **J,** The barplot shows the relative cell viability after OCI-LY1 and Karpas 422 cells were treated with DMSO, YC8-02 (OCI-LY1: 6 μmol/L; Karpas 422: 2 μmol/L), GCN2IN6 (OCI-LY1: 7.5 μmol/L; Karpas 422: 10 μmol/L), and combinations for 72 hours. Cells were subject to flow cytometry for viability tests (DAPI staining) and counting. *, *P* < 0.05; **, *P* < 0.01. NS, not significant. Error bars represent the mean ± SD of three or more replicates.

ATF4 translation is known to be induced by metabolic stress, such as amino acid deprivation (16). The high metabolic demands of DLBCL cells can exhaust nutrients in the culture medium within 48 hours and stimulate ATF4 translation (Fig. 6A). Replenishment with fresh medium to restore nutrients blocked the ATF4 translation, whereas SIRT3-depleted cells neither induced ATF4 translation nor reacted to the nutrient level changes in either medium condition (Fig. 6A). Activation of ATF4 translation was also impaired in SIRT3-deficient cells under glutamine starvation (Fig. 6B and C) but less affected in ER stress induced by tunicamycin. These data suggest that SIRT3 depletion specifically altered the nutrient conditions in DLBCL cells, which in turn interfered with the conditions for ATF4 translation. In contrast, general protein translation as reflected by the yellow fluorescent protein (YFP) reporter was not affected in either nutrient or ER stress conditions (Supplementary Fig. S6A).

Metabolic stress–induced ATF4 translation is normally activated upon depletion of amino acids. However, our metabolic profiling on intracellular amino acids in SIRT3-deficient versus control DLBCL cells revealed a global increase of both essential and nonessential amino acids (EAA and NEAA; Fig. 6D). SIRT3 induces efficient incorporation of glutamine into the TCA cycle, thus creating constant amino acid demand, whereas SIRT3 knockdown blocks glutaminolysis and induces accumulation of glutamine (9). Moreover, autophagy-induced protein degradation can increase cellular amino acids, including glutamine (27, 28). Hence, due to both reasons, amino acid accumulation, especially glutamine, may explain the suppression of ATF4 translation caused by SIRT3 depletion. To test this, we specifically cultured the DLBCL cells in mediums with high levels of l-glutamine, d-glutamine, or d-glucose (as control) to see whether glutamine abundance is responsible for the nutrient sensing effects by ATF4. Notably, l-glutamine significantly reduced ATF4 translation at a late time point of 96 hours, but not at the earlier 48-hour time point where we already see ATF4 activation after nutrient depletion (Supplementary Fig. S6B), suggesting that there may be additional amino acids contributing to control ATF4 translation in DLBCL cells. We therefore cultured DLBCL cells with a supplement of NEAAs, EAAs, l-glutamine, d-glucose, or in combinations. Strikingly, NEAAs and their combinations with other nutrients inhibited the ATF4 translation at earlier time points to similar levels as complete media (Fig. 6E). Administering NEAAs attenuated accumulation of ATF4 protein in multiple DLBCL cell lines regardless of cell of origin, subtypes, or somatic mutations (Fig. 6F).

These data suggest ATF4 translation is highly sensitive to NEAA flux in DLBCL cells. Indeed, SIRT3 depletion induced accumulation of most NEAAs (Fig. 6D). According to our data and the literature (27–29), amino acid accumulation could result from impaired TCA cycle metabolism and autophagy. Thus, restoring the TCA cycle and inhibiting autophagy might be expected to prevent accumulation of amino acids in SIRT3-depleted cells, which in turn might lead to further upregulation of ATF4 (27, 28). Indeed, we showed that DMKG can block autophagy and restore ATF4 protein levels in SIRT3-depleted cells (Fig. 5C). Here, we further observed that DMKG can attenuate most of the amino acid accumulation caused by SIRT3 knockdown (Fig. 6G). Blocking autophagy by depleting ATG7 was also shown to reduce amino acid abundance in starved cells (27), and accordingly we observed that ATG5 depletion in DLBCL and 293T cells was accompanied by greater induction of ATF4 upon glutamine starvation (Fig. 6H and Supplementary Fig. S6C, respectively). Therefore, autophagy plays a major role in cellular amino acid flux, which in turn tunes the activation of ATF4 translation.

### Inhibition of ATF4 Translation Can Enhance the Cell Toxic Effect Induced by SIRT3 Inhibitor

We previously developed a highly active and selective mitochondrial localized SIRT3 inhibitor called YC8-02 (9). Here, we found that treatment with YC8-02 can also decrease the ATF4 protein level while activating autophagy, similar to SIRT3 knockdown (Fig. 6I). Given that ATF4 shRNA also impaired lymphoma cell growth (Fig. 3A), we wondered whether further antilymphoma activity would be gained by also targeting DLBCL cells through inhibition of ATF4 translation. Thus, GCN2 is an ideal candidate, because it is known to phosphorylate EIF2A and induce ATF4 translation upon amino acid depletion (30–32), and GCN2 inhibitors have been proposed as anticancer drugs (16, 33, 34). For example, compound GCN2IN6 was shown to block translation of ATF4 induced by GCN2 activation and metabolic starvation (33, 34). Accordingly, we observed that GCN2IN6 inhibited cell proliferations of DLBCL cell lines (Supplementary Fig. S6D). Moreover, combined treatment with YC8-02 and GCN2IN6 yielded significantly greater antilymphoma effect as compared with either drug alone (Fig. 6J), as also observed when administering increasing doses of YC8-02 to a fixed dose of GCN2IN6 (Supplementary Fig. S6E). Therefore, blocking ATF4 translation can further sensitize DLBCL cells to the effect of SIRT3 targeted therapy.

**Figure 7.**
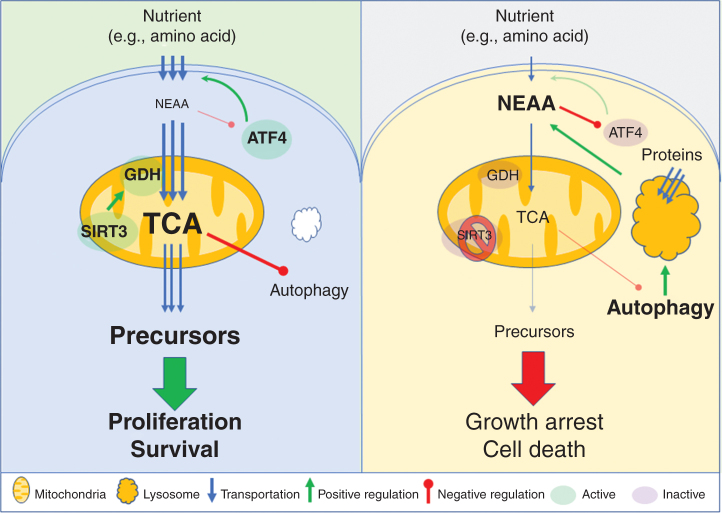
Graphical summary of SIRT3–ATF4 regulation in DLBCLs. Left, DLBCL cells depend on glutamine anaplerosis driven by SIRT3 and GDH to produce metabolic precursors from the TCA cycle for cell survival and proliferation, which also suppress autophagy and the downstream protein recycling in the lysosome. The active proliferation and high metabolic demand of DLBCL cells leads to a shortage of NEAAs and results in translational activation of ATF4, which can transcribe target genes for importation of extracellular nutrients to maintain the amino acid flux. Right, pharmaceutically inhibiting or knocking down SIRT3 suppresses the TCA cycle metabolism as a metabolic engine and decreases the consumption of amino acids (including NEAAs). The reduced TCA cycle metabolism in turn triggers activation of autophagy, which produces amino acids from lysosomal protein degradations to compensate the metabolic suppression. However, the increased amino acids cannot be used in the mitochondria of the defective TCA cycle, but instead block the translation of ATF4 and then shut down the nutrient importation. Together, these induce metabolic stress in DLBCL cells and lead to cell-cycle arrest and death. The larger, bold font indicates more activity or function of indicated proteins or biological activities. The thickness of lines and numbers of arrows indicate the impacts of upstream molecules/biological activities to downstream targets.

Collectively, our data suggest that anaplerotic metabolic homeostasis in DLBCL is critically dependent on a SIRT3–ATF4 functional axis where rapid amino acid flux induced by SIRT3 results in enhanced ATF4 translation to maintain constant availability of amino acids. SIRT3 depletion results in amino acid, especially NEAA, accumulation and compensatory autophagy, which in turn suppresses ATF4 translation due to the global increase in amino acids. This culminates in a vicious circle whereby cells undergo destructive autophagy because they cannot efficiently obtain amino acids from other sources due to downregulation of ATF4 and the amino acids produced through proteolysis cannot be efficiently used for anaplerotic metabolism (Fig. 7).

## DISCUSSION

Herein we identify a critical SIRT3–ATF4 metabolic functional axis for maintenance of proliferation, survival, and anaplerotic metabolism in DLBCL. Suppression of this axis leads to a vicious cycle of destructive autophagy and cell death in these tumor cells. More specifically, DLBCL cells express high levels of SIRT3, which deacetylates (and thus activates) GDH and other anaplerotic enzymes (like IDH2) to promote efficient entry of amino acids such as glutamine into the TCA cycle. This allows DLBCL cells to generate robust quantities of metabolic intermediates such as AcCoA to support cell growth and proliferation and to suppress autophagy with its associated catabolic destruction of cellular proteins and macromolecules (9). DLBCLs are among the most highly proliferative of all tumors and hence are highly dependent on continuous supply and utilization of nutrients such as amino acids (10, 35). Rapid amino acid flux in DLBCL cells can lead to a state of relative amino acid deprivation, which our data suggest triggers translational upregulation of ATF4. Induction of ATF4 target genes involved in amino acid transport and biosynthesis may play a critical role in this context to maintain a steady supply of amino acids to maintain SIRT3-driven anaplerotic metabolism (16, 36).

In the nutrient-deprived environment, cancer cells can survive by cannibalizing themselves via autophagy to recycle macromolecules, such as proteins and DNA, for energy and survival (24, 27, 28). This metabolic change disrupts production of energy and metabolic precursors in the mitochondria, yielding strong induction in autophagy. Although autophagy can restore key metabolic precursors (27, 37) such as glutamine, this effect is futile when glutamine cannot be efficiently utilized in the TCA cycle to generate AcCoA (38), which would otherwise suppress autophagy. Along these lines, our previous findings showed that SIRT3 plays important roles in supporting the metabolic needs of DLBCL cells (9). SIRT3-deficient DLBCL cells manifested global loss of TCA cycle metabolites, such as citrate, α-KG, and AcCoA, caused by impaired entry of glutamine into the TCA cycle (9). To compensate for this loss, DLBCL cells divert glucose utilization from glycolysis (decreased lactate and 3-phosphoglyceric acid) and biosynthesis (decreased serine biosynthesis) to the TCA cycle, which is still not sufficient to rescue DLBCL cells from SIRT3 loss of function (9). Most critically, both SIRT3 inhibition and autophagy activation led to accumulation of amino acids that results in impaired translation of ATF4. However, these amino acids do not rescue the TCA cycle defects in the absence of SIRT3 function. For example, branched-chain amino acids (BCAA) may also be used in the TCA cycle, and we observed that BCAAs accumulated in SIRT3-depleted cells (Fig. 6D; Supplementary Fig. S6F), possibly due to autophagy activation. Their downstream metabolites (isovalerylcarnitine, 2-methylbutyroylcarnitine, and isobutyryl-L-carnitine) did not increase, which didn't support BCAA metabolism as the alternative source for TCA cycle metabolism. Consequently, DLBCL cells lose a critical “metabolic plasticity” rescue mechanism and undergo proliferation arrest and cell death.

The links between autophagy and ATF4 can be quite complex. For example, ATF4 can induce transcriptional activation of certain autophagy-related genes and thus facilitate induction of autophagy (39). On the other hand, autophagy may downregulate ATF4 by reducing unfolded proteins via ER-associated degradation (ERAD) in ER stress conditions (40). Here, we show that in DLBCL cells, autophagy represses ATF4 accumulation through modulating cellular amino acid levels. In previous studies, there are clues suggesting that autophagy might negatively regulate ATF4 signaling. Indeed, we observed enrichment for the ATF4 activation signature in proteomic data from Atg5 knockout MEFs (24). Similarly, autophagy-deficient (ATG3/ATG7 knockout) HCT116 cells were reported to feature elevated ATF4 target genes in starvation conditions (36). Notably, the effect of autophagy on intracellular amino acids may have a significant impact in regulating ATF4 abundance. Autophagy is an important source of amino acids for certain cancer cells (28). For example, lung cancer cells rely on autophagy to regenerate amino acids, especially glutamine, to support malignant cell proliferation (27, 37). However, SIRT3-depleted DLBCL cells undergo a powerful autophagy response and manifest global increase of amino acid, which together with decreased amino acid flux into the TCA cycle can effectively inhibit ATF4 translation that would normally be induced by amino acid deprivation. Altogether, these data suggest that autophagy can tightly control ATF4 activation in accordance with cellular amino acid levels.

Our results underline that SIRT3 effects can be highly cancer cell context specific (11), a point that is nicely illustrated by the lack of effect of SIRT3 depletion on HIF1α functions in DLBCL cells. Given that both HIF1α and ATF4 are activated by distinct forms of metabolic and cellular stress, these data further point to how distinct tumor types are dependent on different types of stress signaling. For example, studies in murine Eµ-MYC pro–B-cell leukemia/lymphomas suggested that ATF4 is induced by ER stress (41) and MYC-induced nutrient exhaustion (42). However, in human DLBCLs (where MYC translocations are relatively uncommon), it is evident that SIRT3 is critical to drive the nutrient flux to the TCA cycle, with ATF4 playing an essential role by maintaining a constant supply of amino acids as fuel to support anaplerotic metabolism, independent of genetic background and disease subtype. This symbiotic relationship between SIRT3 and ATF4 underlines a novel functional axis between these mitochondrial and nuclear proteins that delineates a crucial metabolic vulnerability in these tumors.

## METHODS

### Human Tonsil and DLBCL Samples

The same samples were collected and used in our previous study (9). Briefly, primary human deidentified leftover tonsil samples and DLBCL samples were obtained and processed under the Institutional Review Board protocols of Weill Cornell Medicine (New York, NY; #0804009762) and the University of Turin (Turin, Italy; #0081521) after written informed consent, in accordance with the Declaration of Helsinki protocol. Human B-cell populations were affinity purified using standard protocols, and GC B-cell purity was determined by flow cytometry analysis of surface logD (BD Pharmingen), CD77 (AbD Serotech), and CD38 (BD Pharmingen; ref. 43).

### Mice

The Research Animal Resource Center of Weill Cornell Medicine approved all mouse procedures. All mice were bred and housed on a 12-hour light/dark cycle. *Sirt3*^−/−^ and 129S1 mice were purchased from The Jackson Laboratory. *VavP-Bcl2* (44), and *Sirt3*^−/−^ mice were crossed to generate *VavP-Bcl2;Sirt3*^−/−^ and *VavP-Bcl2;Sirt3*^+/+^ mice as donor mice for bone marrow transplantation. Six-month or older *VavP-Bcl2;Sirt3*^−/−^ and *VavP-Bcl2;Sirt3*^+/+^ mice were sacrificed for evaluation of ATF4 protein levels. Mice with splenomegaly phenotypes were recognized as mice bearing lymphoma. Splenocytes were collected after red blood cell lysis, counted, and lysed for Western blot analysis.

### Cell Lines and Antibodies

The DLBCL cell lines, OCI-LY1 (Ontario Cancer Institute, OCI), were grown in Iscove's medium supplemented with 10% FBS and penicillin G/streptomycin; HBL-1 (gifted from Jose A. Martinez-Climent, Universidad de Navarra, Pamplona, Spain), TMD8 (gifted from Louis M. Staudt, NCI, Bethesda, MD), Pfeiffer (ATCC), and Karpas 422 and SU-DHL-4 (Deutsche Sammlung von Mikroorganismen und Zellkulturen, DSMZ) were grown in RPMI medium supplemented with 10% FBS, penicillin G/streptomycin, l-glutamine, and HEPES. HCT116 and HEK-293T cell (ATCC) lines were cultured in DMEM supplemented with 10% FBS and 1% penicillin G/streptomycin. All cells were validated by the University of Arizona Genetics Core before being used. Cell lines were tested with MycoAlert PLUS Mycoplasma Detection Kit (Lonza #LT07-705) or cultured with PlasmocinTM Prophylactic (InvivoGen) to prevent *Mycoplasma* contamination. Cells were usually used for experiments within 15 passages after being thawed.

Antibodies of SIRT3 (5940S), ATF4 (11815S), ATG5 (12994S), LC3I/II (12741S), EIF2A (5324P), phospho-EIF2A (3398S), Grp75 (3593S), p62 (5114S), and acetylated histone 3 (8848S) were purchased from Cell Signaling Technology. The histone 3 (09-838) antibody was from Millipore, the GDH (14299-1-AP) antibody was from Proteintech Group, and the ACTB (A5441-2ML) antibody was from Sigma-Aldrich. GCN2IN6 (HY-130240) was purchased from MCE (MedChemExpress).

### Generation of Lentiviruses

Briefly, the pLKO.1 lentiviral expression vector containing the puromycin resistance gene was used to coexpress individual shRNAs with YFP. shRNA constructs of scramble control and against SIRT3 mRNA were reported in our previous study (9). ATF4 shRNA constructs (shATF4-1 and shATF4-2) target human ATF4 mRNAs at 5′-GCCTAGGTCTCTTAGATGATT-3′ and 5′-GCCAAGCACTTCAAACCTCAT-3′, respectively. pLKO.1 was further modified for rescue experiments by inserting sequences containing ATF4-IRES-GFP. The ATF4-5′UTR-GFP reporter was generated by conjugating 5′-UTR of ATF4 mRNA (cloned from Addgene clone #115969) with GFP and inserting it into pLKO.1 vector with scramble control or SIRT3 shRNAs. The lentiviruses were generated by coexpressing VSV-G and delta-8.9 in HEK-293T cells, and then concentrated using PEG-it (System Biosciences). Cells were infected and cultured for at least 3 days before adding puromycin for selection and various biological assays.

### Cell Lysis and Immunoblotting

Normal GC B cells, DLBCL cell lines, or primary DLBCL specimens were lysed using RIPA lysis buffer containing complete protease inhibitor cocktail to prepare whole-cell lysate. Whole-cell lysates were resolved by SDS-PAGE, transferred to polyvinylidene difluoride membrane (Bio-Rad), and probed with the indicated primary antibodies: anti-SIRT3, anti-ATF4, anti-GRP75, anti–acetylated histone 3, anti-LC3, and anti-p62, purchased from Cell Signaling Technology, and anti-ACTB, anti–histone 3, and anti-GDH, purchased from Sigma-Aldrich, Millipore, and Proteintech Group, respectively. Membranes were then incubated with a corresponding peroxidase-conjugated secondary antibody. Protein signals were detected using enhanced chemiluminescence. Densitometry values were obtained by using ImageJ 1.44o.

### Cell Proliferation and Viability Analyses

The methods for these experiments were described in our previous study (20). DLBCL cell lines were infected with lentiviruses carrying control, SIRT3, or ATF4 shRNAs with YFP. Dead cells in control and SIRT3-depleted cells were stained with DAPI (1 μg/mL), and percentages of viable cells (DAPI^−^) were quantified by flow cytometry. The change in the percentage of YFP^+^ population was calculated by normalizing to the first time point (5 days after infection). For growth curves, Karpas 422, OCI-LYI, and HBL-1 cells expressing control or SIRT3 shRNAs were counted over a period of 7 days by trypan blue exclusion. YFP^+^ and YFP^−^ cells were cocultured, and the dilution of the fluorescent dye in YFP^+^ and YFP^−^ cells was monitored after 5 days by flow cytometry. The data were analyzed using FlowJo software.

### ATF4 Translation Reporter Assay

The ATF4-5′UTR-GFP reporter (22) was generated by conjugating 5′-UTR of ATF4 mRNA with GFP and inserting it into pLKO.1 vector with scramble control or SIRT3 shRNAs. Karpas 422 and OCI-LY1 cell lines were infected with these ATF4 translation reporters, and their GFP intensities were monitored with flow cytometry at different time points after infection. The data were analyzed using FlowJo software, and mean fluorescence intensities (MFI) were obtained for quantifications. In Fig. 2G and H, data were analyzed by normalizing to MFI from cells expressing YFP without 5′-UTR of ATF4 to avoid basal variations of ATF4 translation. In Fig. 6B and D, cells were treated at day 4 after infection in glutamine-depleted RPMI medium or with tunicamycin (10 μg/mL) for 15 to 16 hours. MFIs of ATF4-5′UTR-GFP were measured with flow cytometry. The FCs of MFIs were calculated with intensities of ATF4-5′UTR-GFP from treated cells over untreated cells.

### Metabolomic Profiling

The metabolomic studies were done as in our previous study (9). Briefly, Karpas 422 cells were infected with viral vectors containing control or SIRT3 shRNAs. Cells were collected from day 6 after infections after puromycin selection. Cell pellets were flash frozen by dry ice and shipped to Metabolon for nontargeted metabolomic profiling. Metabolomic data were obtained from Metabolon through untargeted metabolomic profiling using DLBCL cell lines. Data were preprocessed to exclude metabolites that contain missing values for ≥20%, and metabolite abundances were log transformed and quantile normalized. Data of 20 amino acids were extracted from all data and plotted as in Fig. 6D.

Metabolic data showed in Fig. 6G were generated from LC/MS. Metabolites were extracted from cell culture with 80% methanol. Metabolite measurement was conducted by LC/MS using a quadrupole-orbitrap mass spectrometer (Q Exactive, Thermo Fisher Scientific) coupled to hydrophobic interaction chromatography via electrospray ionization. The LC separation was done with an XBridge BEH Amide column (150 mm × 2.1 mm, 2.5 mm particle size, 130 A pore size, Waters). Polarity switching mode was used for detection of metabolites in both negative and positive ionization modes, with scan window of m/z 70 to 1,000 at 1 Hz and 70,000 resolution. Solvent compositions were as follows: Solvent A was 20 mmol/L ammonium acetate, 20 mmol/L ammonium hydroxide in 95:5 water:acetonitrile, and pH 9.45, and solvent B was pure acetonitrile. Flow rate was 150 mL/minute, and LC gradient used was as follows: 0 minutes, 85% B; 2 minutes, 85% B; 3 minutes, 80% B; 5 minutes, 80% B; 6 minutes, 75% B; 7 minutes, 75% B; 8 minutes, 70% B; 9 minutes, 70% B; 10 minutes, 50% B; 12 minutes, 50% B; 13 minutes, 25% B; 16 minutes, 25% B; 18 minutes, 0% B; 23 minutes, 0% B; and 24 minutes, 85% B. Injection volume of 10 μL was used. Data were extracted using the MAVEN or El-MAVEN (Elucidata) software (45). Abundance of amino acids was normalized to cell numbers, and cell volumes were evaluated with flow cytometry (46); FCs were calculated by normalizing against values from control cells.

### RNA-seq and Analyses

Total RNA was extracted from DLBCL cell lines expressing control or SIRT3 shRNAs at day 8 after infection using TRIzol (Life Technologies) and RNeasy isolation Kit (Qiagen). RNA concentration was determined using Qubit (Life Technologies), and integrity was verified using Agilent 2100 Bioanalyzer (Agilent Technologies). Libraries were generated using the TruSeq RNA sample kit (Illumina). First-strand synthesis was performed using random oligos and SuperscriptIII (Invitrogen). After second-strand synthesis, a 200-bp paired-end library was prepared following the Illumina paired-end library preparation protocol. Pair-end sequencing (PE50) was performed on Illumina HiSeq2000. RNA-seq results were aligned to hg19 using STAR (47) and annotated to RefSeq using the Rsubread package (48). Unsupervised hierarchical clustering was performed using Euclidean distance and Ward's minimum variance method for the top variable genes within each cell line (95th percentile according to SD). Differential expression was determined using edgeR package glmFit function, correcting for cell line using an additive design model (49, 50). Pathway enrichment of differential expression signatures was calculated using hypergeometric test.

### GSEA

GSEA was done with tools downloaded from the GSEA website (51, 52). Gene lists were either downloaded from the Molecular Signatures Database (MSigDB) or summarized from previous publications (17, 42). Preranked data for cell line GSEA were calculated as the log_2_ ratio of the mean expression among replicates (counts per million). Pre-ranked data for primary cases and GC B cells were calculated as the log_2_ ratio of the mean expression of RMA value (data from accession GSE12195; ref. 53).

### Statistical Analyses

A two-tailed Student *t* test was used to determine the statistical significance of the results. *P* ≤ 0.05 was considered statistically significant. Correlations were determined using Pearson correlation coefficients using GraphPad Prism. Log-rank tests ≤0.05 were standard for significance between subgroups of patients or animals.

### Data Availability

RNA-seq data from this article will be deposited in the Gene Expression Omnibus database. The accession number for the RNA-seq data of DLBCL cells with control or SIRT3 shRNAs is GSE158833.

## Authors' Disclosures

L. Cerchietti reports grants from Leukemia & Lymphoma Society during the conduct of the study, as well as grants from Celgene, NCI, and Leukemia & Lymphoma Society outside the submitted work. H. Lin reports grants from Falk Medical Research Foundation during the conduct of the study, as well as other support from Sedec Therapeutics outside the submitted work. A.M. Melnick reports grants from Falk Foundation during the conduct of the study; grants from Janssen, Epizyme, Sanofi, and Daiichi Sankyo and personal fees from Janssen, Epizyme, AstraZeneca, Bristol Myers Squibb, Daiichi Sankyo, and Exo Therapeutics outside the submitted work; and a patent for 8635-01-US issued. No disclosures were reported by the other authors.

## Authors' Contributions

**M. Li:** Conceptualization, formal analysis, funding acquisition, validation, investigation, visualization, methodology, writing–original draft, project administration, writing–review and editing. **M.R. Teater:** Data curation, software, formal analysis, validation, investigation, writing–original draft. **J.Y. Hong:** Validation, investigation, methodology. **N.R. Park:** Investigation, writing–original draft. **C. Duy:** Conceptualization, investigation, methodology. **H. Shen:** Investigation, methodology. **L. Wang:** Investigation. **Z. Chen:** Data curation, investigation, methodology. **L. Cerchietti:** Conceptualization, resources, investigation. **S.M. Davidson:** Formal analysis, supervision, methodology. **H. Lin:** Conceptualization, supervision, funding acquisition, writing–original draft. **A.M. Melnick:** Conceptualization, resources, supervision, funding acquisition, writing–original draft, project administration, writing–review and editing.

## Supplementary Material

Supplementary Data

Supplementary Data
