# Supplementary material for: Translational Activation of ATF4 through Mitochondrial Anaplerotic Metabolic Pathways Is Required for DLBCL Growth and Survival
Source: Blood Cancer Discov. 2021 Nov 9;3(1):50–65. doi: 10.1158/2643-3230.BCD-20-0183 (PMC9789686; doi:10.1158/2643-3230.BCD-20-0183)
Supplement: Supplementary Data [file bcd-20-0183_supp1-6.pdf]

Supplementary Figure S1

A

NES	Karpas 422		HBL1		LY1	
Signatures	sh1	sh2	sh1	sh2	sh1	sh2
ATF6	0.453	-0.536	-0.863	1.116	-0.495	-0.512
CHOP	-1.036	-1.025	-1.441	-0.901	1.273	1.100
XBP1	0.495	0.798	0.652	1.679	-0.935	-0.620

FDR	Karpas 422		HBL1		LY1	
Signatures	sh1	sh2	sh1	sh2	sh1	sh2
ATF6	1.000	0.990	0.693	0.249	1.000	0.990
CHOP	0.368	0.419	0.021	0.655	0.110	0.296
XBP1	0.998	0.839	0.957	0.001	0.577	0.979

B

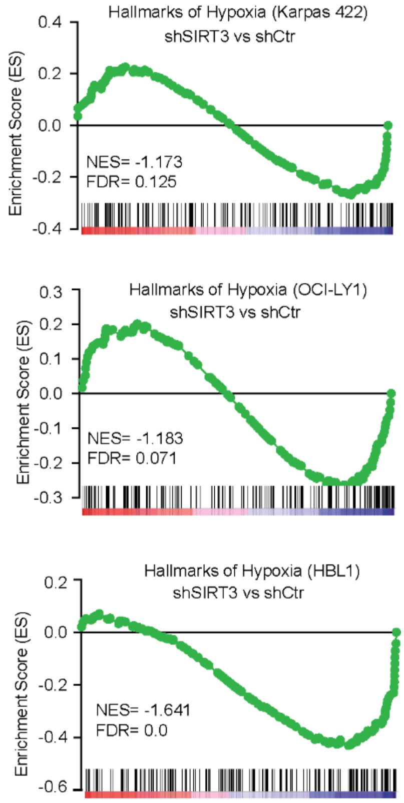

C

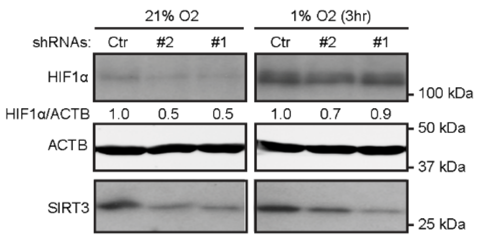

D

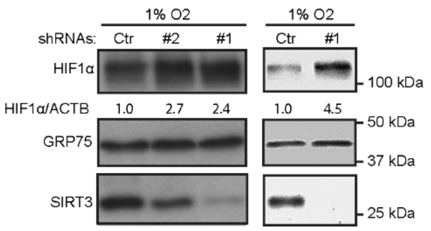

Supplementary Figure S1, **(A)** Tables showed the NES (normalized enrichment score) and FDR (false discovery rate) values from GSEA analysis on RNA sequencing data from DLBCL cell lines (Karpas 422, HBL1 and OCI-LY1) with indicated signatures (ATF6, CHOP and XBP1). Positive or negative values of NES represent positive and negative enrichment, respectively. FDR <0.05 shows significant enrichment in the NES table. **(B)** GSEA results show that HIF1a target genes are not enriched in SIRT3 regulated genes in different DLBCL cells lines. Genes of signature as hallmarks of hypoxia (from molecular signature database on GSEA website) was used as HIF1a target gene set. **(C)** Western blots show the HIF1a protein level in HEK-293T and HCT-116 cells in control or SIRT3 knock down cells in hypoxia condition for 3 hr (1% oxygen). Protein levels of HIF1a were quantified with densitometry results normalized

to ACTB levels. **(D)** Western blots show HIF1a protein level in Karpas 422 cells in control or SIRT3 knock down cells with shRNAs in lentiviral vectors. Experiments were down in both normoxia (21% oxygen) or hypoxia condition for 3 hr (1% oxygen). Protein levels of HIF1a were quantified with densitometry results normalized to ACTB levels.

Supplementary Figure S2

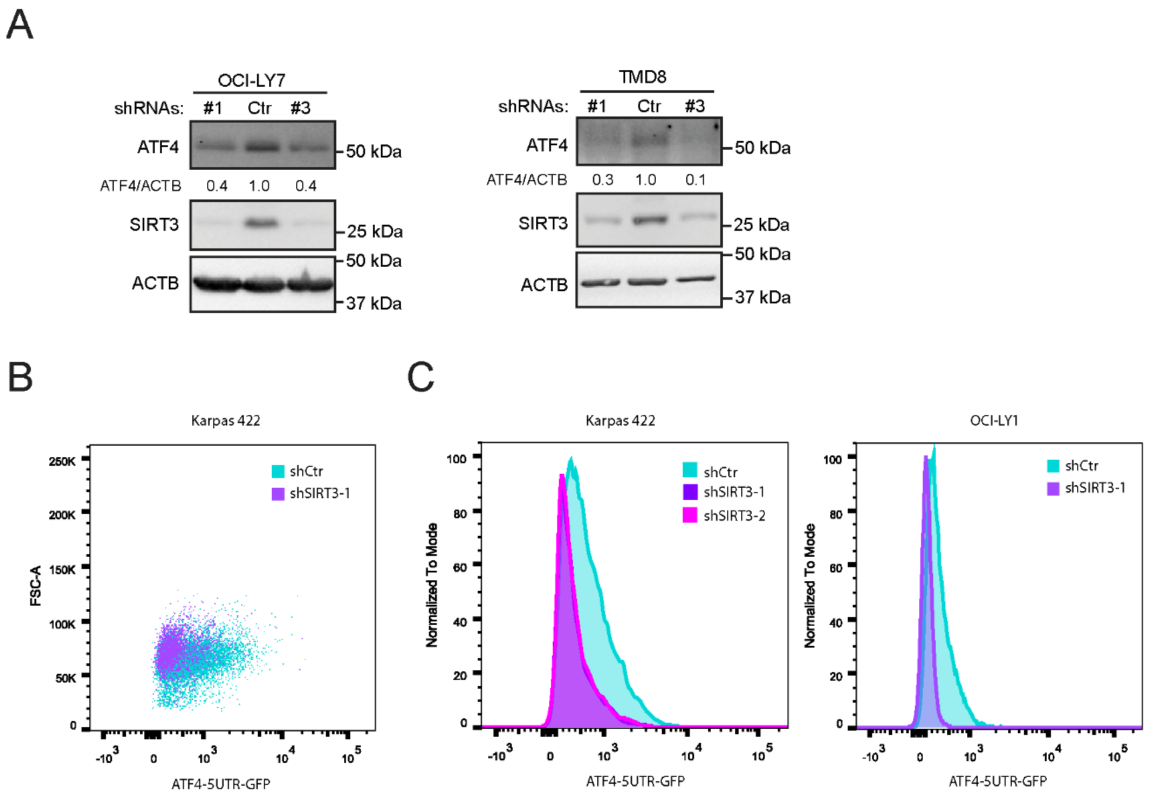

Supplementary Figure S2, (A) Western blots show ATF4 protein levels in different DLBCL cells with control or SIRT3 shRNAs. SIRT3 was blotted showing knocking down efficiency and ACTB was used as reference protein control. The changes of protein levels were quantified with densitometry results. **(B)** Representative flow plot for ATF4 translational reporter expressed in Karpas 422 cells with control or SIRT3 shRNAs. **(C)** Histograms showing the staining intensities of the ATF4 translational reporter from DLBCL cells expressing control or SIRT3 shRNAs.

Supplementary Figure S3

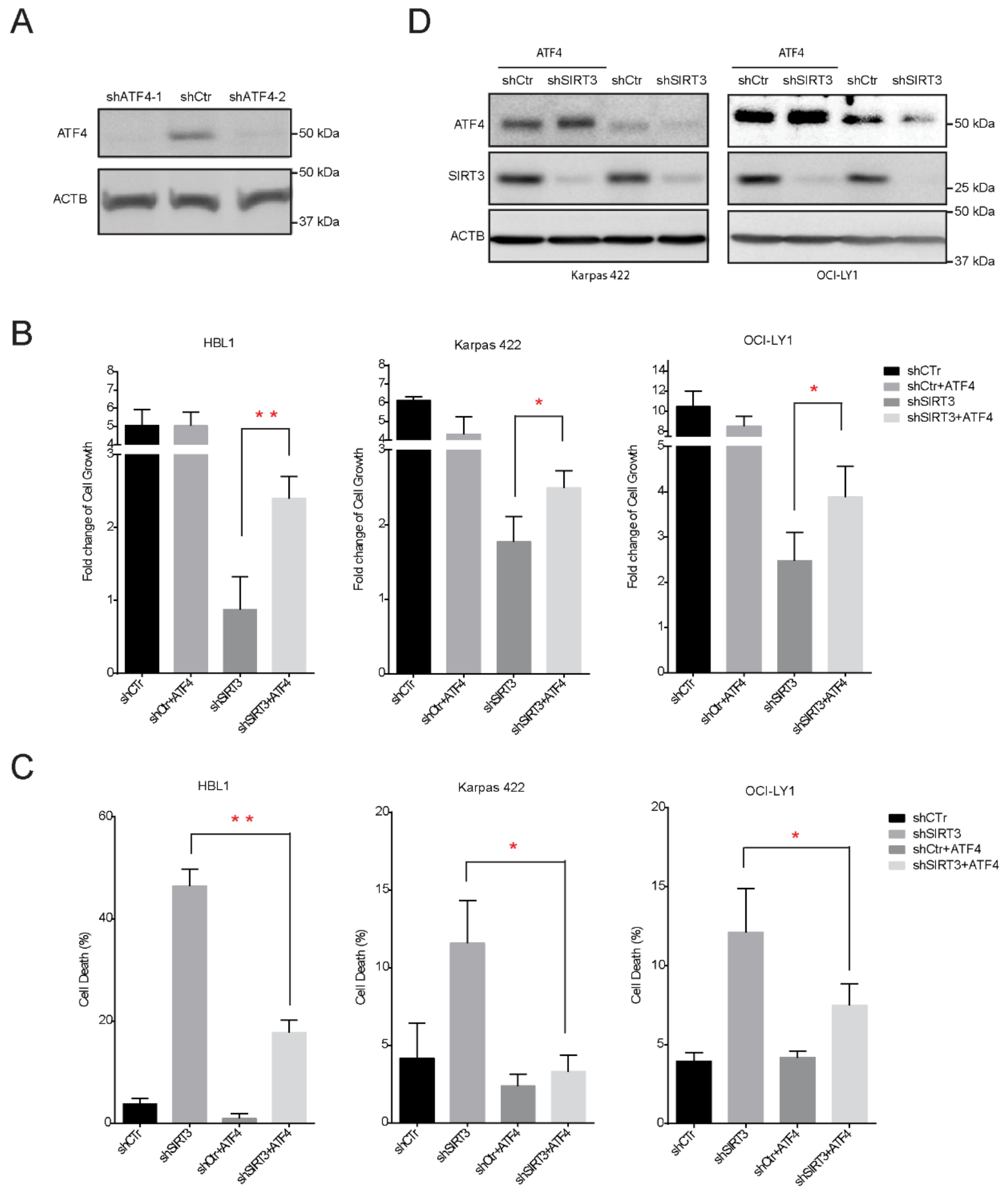

Supplementary Figure S3, **(A)** Western blot results show the knocking down efficiency of ATF4 shRNAs. Karpas 422 cells were transduced with control or SIRT3 shRNAs and collected for Western blots. **(B)** Effects of ATF4 overexpression on cell proliferation (numbers) in control or SIRT3 knockdown cells. The Y axis shows fold changes calculated with cell numbers at end time point normalized to those of initial time point in each condition. **(C)** Effects of ATF4 overexpression on cell death caused by SIRT3 shRNAs. The Y axis shows the percentage of dead cell from flow cytometry

by staining with DAPI. **(D)** Western blots show exogenous ATF4 expression in DLBCL cells with control or SIRT3 shRNAs. The cells were infected with lenti-vectors expressing human ATF4 cDNA and SIRT3 or control shRNAs. The cells were cultured for 5-6 days post infection and then processed for immunoblots with the indicated antibodies. * $p<0.05$, ** $p<0.01$ show the statistical significance of repetitive experiments.

Supplementary Figure S4

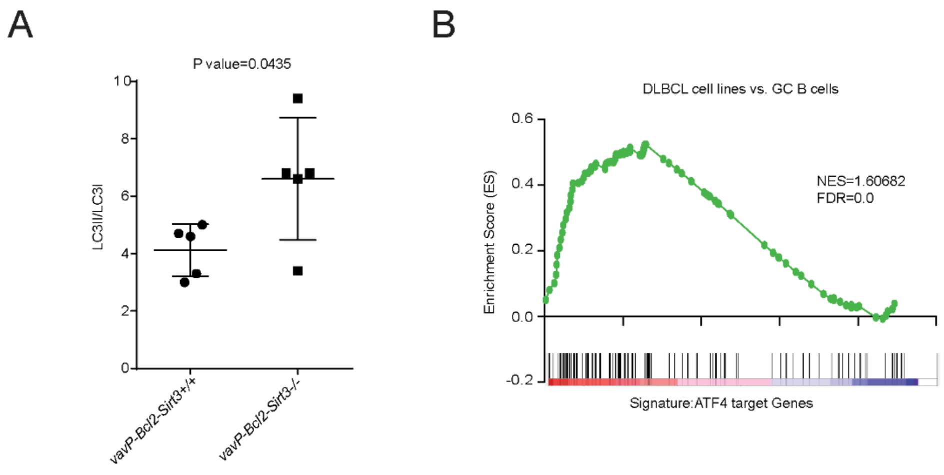

Supplementary Figure S4, **(A)** Summarized results of autophagy levels (evaluated by densitometry results of LC3II/LC3I) in splenocytes from *vavP-Bcl2;Sirt3*$^{+/+}$ and *vavP-Bcl2;Sirt3*$^{-/-}$ mice. **(B)** GSEA analysis shows the enrichment of ATF4 target genes in a group of DLBCL cell lines vs. normal GC B cells.

Supplementary Figure S5

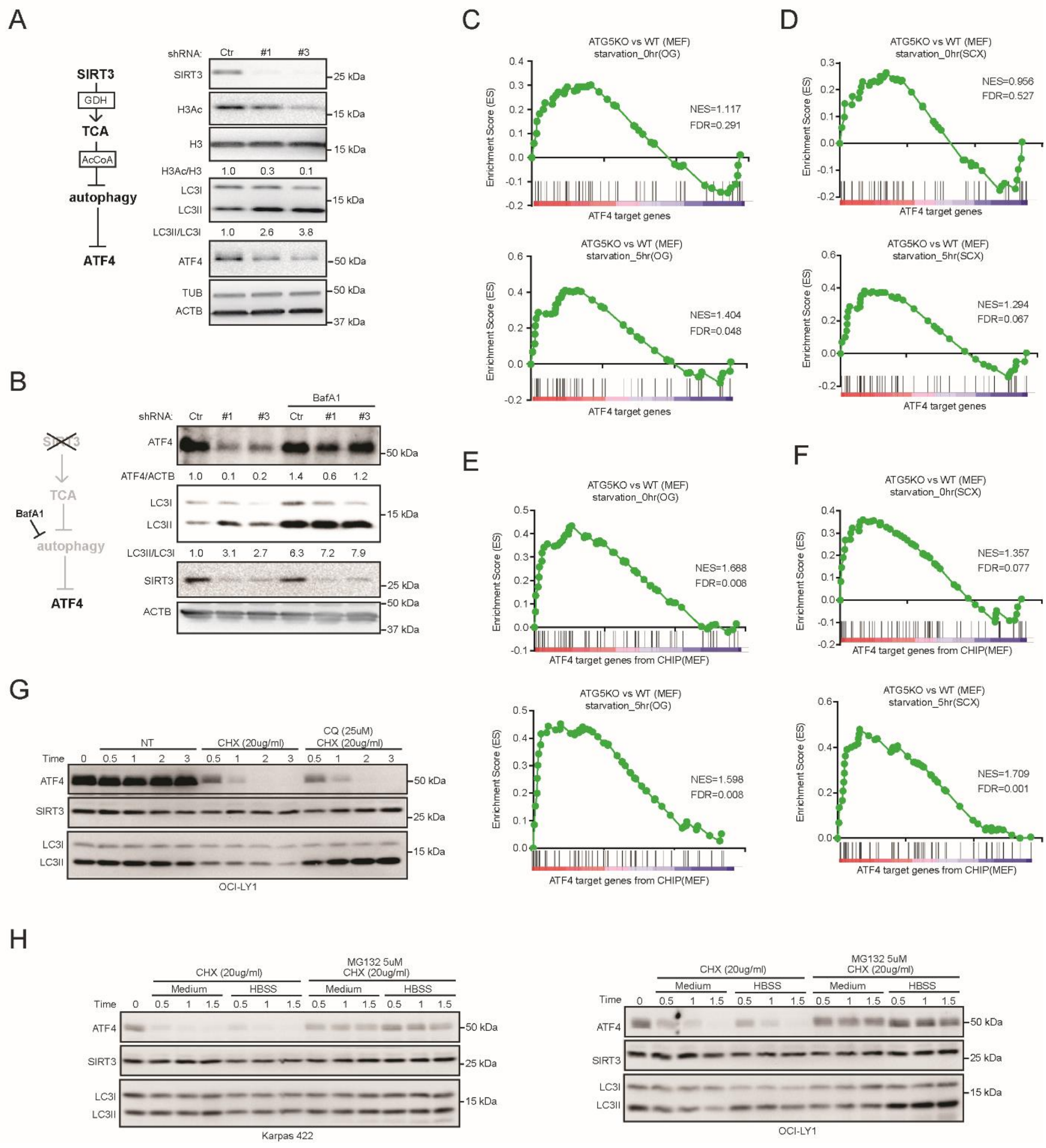

Supplementary Figure S5, **(A)** Western blots show changes of ATF4, H3Ac, LC3 and other protein levels in Karpas 422 cells expressing control or SIRT3 shRNAs. Protein level changes were quantified with densitometry results. On the right, hypothetical cascade model was presented. **(B)** Western blots show the ATF4 protein level being rescued by BafA1 (100nM) in SIRT3 knocked down Karpas 422 cells. Treatment was done in 16-18 hours, followed by immunoblot with the indicated antibodies.

Densitometry values are shown for ATF4/ACTB and LC3II/LC3I rations. On the right, hypothetical cascade model was presented. GSEA analysis was done with published proteomic data using the ATF4 target genes list from ATF4 CHIP in MEF cells. Proteomic results were from SILAC analysis coupled with OG**(C)** or SCX methods **(D).** Upper panel showed analysis of comparing ATG5 KO MEF vs. wildtype MEF. Lower panel showed analysis of comparing ATG5 KO MEF vs. wildtype MEF in HBSS starvation condition for 5 hours. **(E)** & **(F)** similar analysis were done with gene signatures of ATF4 target genes list from ATF4 CHIP in MEF cells. **(G)** Western blots show ATF4 protein levels under CHX and CQ treatment in OCI-LY1 cells. Protein samples were collected at the indicated time points after treatment to monitor the kinetics of ATF4 degradation. SIRT3 and LC3 were blotted as controls. **(H)** Western blots show ATF4 protein levels under CHX and MG132 treatment in Karpas 422 and OCI-LY1 cells. Protein samples were collected at the indicated time points after treatment to monitor the kinetics of ATF4 degradation. Cells were treated in both culture medium and HBSS (starvation condition). SIRT3 and LC3 were blotted as controls.

Supplementary Figure S6

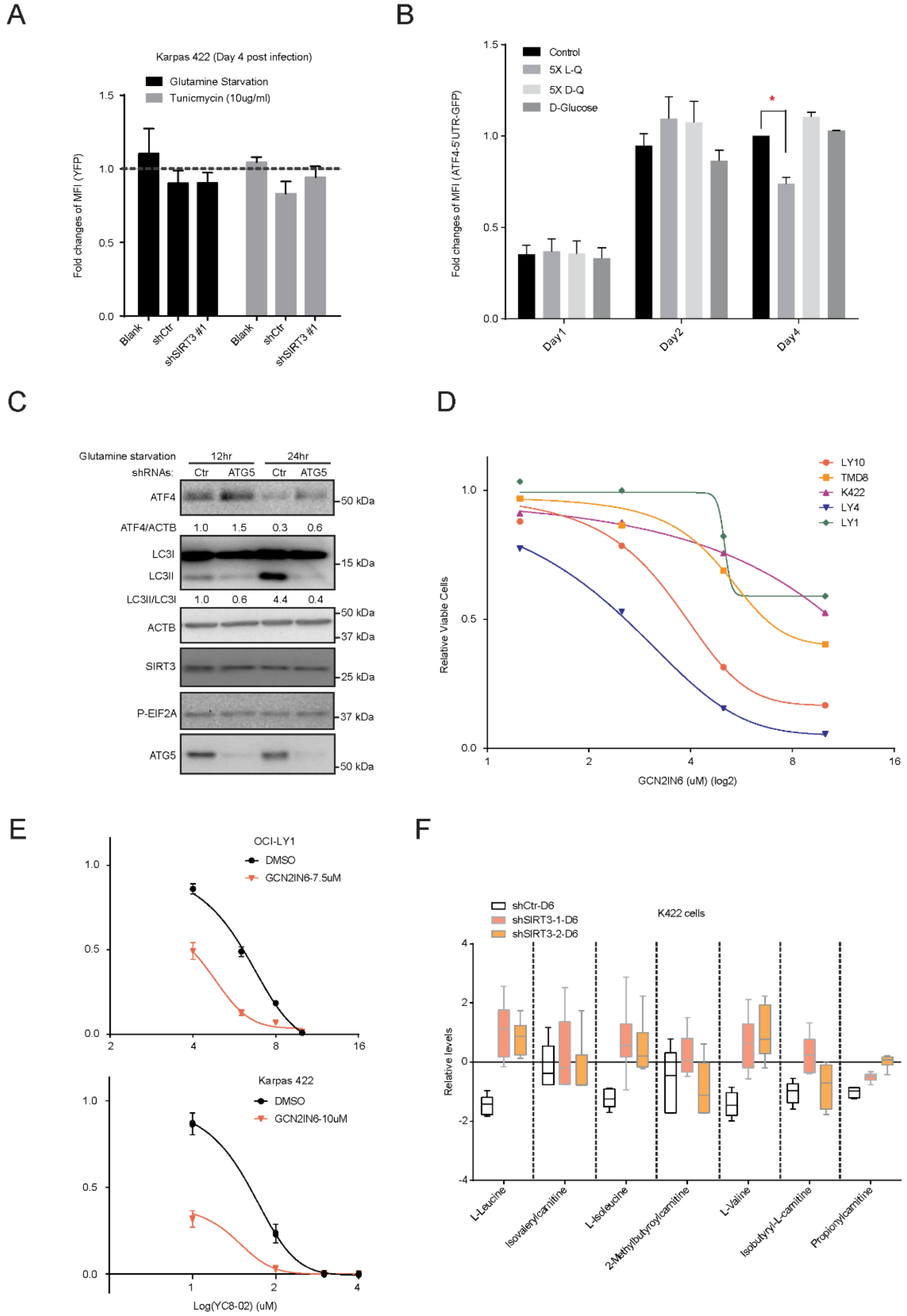

Supplementary Figure S6, **(A)** Fold changes of MFI of YFP reporter without ATF4 5'UTR expressed in Karpas 422 cells with control or SIRT3 shRNAs under treatment conditions as in Figure 6B. **(B)** ATF4 translation activities from Karpas 422 cells expressing the ATF4 translational reporter and being cultured in fresh medium with 10mM L-Q, 10mM D-Q, or 20mM D-glucose or without supplements (control). ATF4 translation reporter activities were monitored with flow cytometry at the indicated

timepoints after the culture conditions were set up. MFI was used to quantification and normalized to the activities from Day 2 control cells. **(C)** Western blots results show ATF4 level changes in control or ATG5 knock down 293T cells under glutamine starvation condition. Samples were collected at different time points and protein level changes were quantified with densitometry results. **(D)** Dose-response curves for the indicated DLBCL cell lines exposed to increasing concentrations of GCN2IN6 for 72 hours. Cell tilter Glo was used to quantify the cell numbers after treatment. **(E)** Dose response curves show the relative cell viability for DLBCL cell lines treated with YC8-02, GCN2IN6 or the combination. GCN2IN6 was used at a fixed dose for OCI-LY1 (7.5uM) and Karpas 422(10uM) in. **(F)** Box plot shows relative levels (log2 transformed and scaled) of metabolites from branched chain amino acids metabolism. Karpas 422 cells were infected with control or SIRT3 shRNAs and cultured for 6 days and then profiled by LC/MS. Data were summarized from 5-6 replicate samples for each condition. *p value<0.05, **p value<0.01. Error bars represent the mean +/- SD of three or more replicates.
